# Silencing of omega-5 gliadins in transgenic wheat eliminates a major source of environmental variability and improves dough mixing properties of flour

**DOI:** 10.1186/s12870-014-0393-1

**Published:** 2014-12-24

**Authors:** Susan B Altenbach, Charlene K Tanaka, Bradford W Seabourn

**Affiliations:** USDA-ARS, Western Regional Research Center, 800 Buchanan Street, Albany, CA 94710 USA; USDA-ARS, Center for Grain and Animal Health Research, Hard Winter Wheat Quality Laboratory, 1515 College Avenue, Manhattan, KS 66502 USA

**Keywords:** Environment, Fertilizer, Food allergens, Gliadins, Gluten proteins, RNA interference, Transgenic plants, Wheat flour quality

## Abstract

**Background:**

The end-use quality of wheat flour varies as a result of the growth conditions of the plant. Among the wheat gluten proteins, the omega-5 gliadins have been identified as a major source of environmental variability, increasing in proportion in grain from plants that receive fertilizer or are subjected to high temperatures during grain development. The omega-5 gliadins also have been associated with the food allergy wheat-dependent exercise-induced anaphylaxis (WDEIA). Recently, transgenic lines with reduced levels of omega-5 gliadins were developed using RNA interference (RNAi). These lines make it possible to determine whether changes in the levels of omega-5 gliadins in response to environmental conditions and agronomic inputs may be responsible for changes in flour end-use quality.

**Results:**

Two transgenic wheat lines and a non-transgenic control were grown under a controlled temperature regimen with or without post-anthesis fertilizer and the protein composition of the resulting flour was analyzed by quantitative two-dimensional gel electrophoresis (2-DE). In one transgenic line, all 2-DE spots identified as omega-5 gliadins were substantially reduced without effects on other proteins. In the other transgenic line, the omega-5 gliadins were absent and there was a partial reduction in the levels of the omega-1,2 gliadins and the omega-1,2 chain-terminating gliadins as well as small changes in several other proteins. With the exception of the omega gliadins, the non-transgenic control and the transgenic plants showed similar responses to the fertilizer treatment. Protein contents of flour were determined by the fertilizer regimen and were similar in control and transgenic samples produced under each regimen while both mixing time and mixing tolerance were improved in flour from transgenic lines when plants received post-anthesis fertilizer.

**Conclusions:**

The data indicate that omega-5 gliadins have a negative effect on flour quality and suggest that changes in quality with the growth environment may be due in part to alterations in the levels of the omega gliadins. Because a known food allergen and one of the major sources of environmentally-induced variation in wheat flour protein composition has been eliminated, the transgenic lines may yield flour with both improved end-use quality and more consistent functionality when grown in different locations.

**Electronic supplementary material:**

The online version of this article (doi:10.1186/s12870-014-0393-1) contains supplementary material, which is available to authorized users.

## Background

The growth conditions of the wheat crop cause changes in the protein composition of the grain as well as variations in the functional properties and allergenic potential of the resulting flour. However, it is difficult to establish links between changes in specific proteins and flour quality because of the complexity of the major wheat flour proteins. The main determinants of flour end-use quality, the gluten proteins, consist of gliadins and glutenins that together comprise about 70-80% of the flour protein. Both types of proteins contain repetitive sequences and an abundance of glutamine and proline and are largely insoluble in aqueous solutions [1,2]. The gliadins are present as monomers in the flour and contribute extensibility to wheat flour dough. These proteins are separated into alpha, gamma and omega gliadin subgroups, each with distinct primary sequences. The glutenins contribute elasticity to dough and consist of two types of proteins, the high-molecular-weight glutenin subunits (HMW-GS) and the low-molecular-weight glutenin subunits (LMW-GS), linked by disulfide bonds into complex polymers with molecular weights that can exceed one million [3]. In addition, some proteins with sequences very similar to alpha, gamma and omega gliadins are incorporated into the polymer. While traditional alpha and gamma gliadins contain six or eight cysteine residues that are involved in intramolecular bonds and omega gliadins do not contain any cysteine, these gliadin-like proteins contain an additional cysteine residue that enables them to be linked to the polymer. It has been hypothesized that these proteins act as terminators of the polymer chain and decrease its size, thereby influencing quality [4,5]. The gliadin-like proteins are sometimes referred to as C- and D-type LMW-GS on the basis of their functional properties. Alternately, they may be referred to as chain-terminating gliadins on the basis of their primary sequences.

Each of the gliadin and glutenin groups contains many similar proteins that also vary among wheat cultivars. Despite this complexity, a detailed map of the flour proteome in which most of the major flour proteins were linked to specific gene sequences was developed for the hard red spring wheat Butte 86 using two-dimensional gel electrophoresis (2-DE) combined with tandem mass spectrometry (MS/MS) [6]. Using this proteomic map, the effects of post-anthesis fertilizer and high temperature on the accumulation of individual flour proteins were investigated in a series of controlled growth experiments [7,8]. These studies demonstrated that, among the gluten proteins, the omega gliadins show some of the largest responses to the application of fertilizer and high temperatures during the period of grain development. Other studies have shown that the omega gliadins also respond to sulfur deficiency [9].

The omega gliadins consist of two subgroups of proteins with distinct sequences, referred to as omega-5 and omega-1,2 gliadins. The omega-5 gliadins have N-terminal sequences beginning with SRL and the repetitive motifs FPQQQ and QQIPQQ. These proteins are of additional interest because of their involvement in the serious food allergy wheat-dependent exercise-induced anaphylaxis (WDEIA) [10,11]. In comparison, the omega-1,2 gliadins have N-terminal sequences beginning with ARE, ARQ or KEL and a different repetitive motif, PQQPFP. Omega-5 chain-terminating gliadins that contain single cysteine residues near the carboxyl termini have been described [12]. However, there is no evidence from analysis of expressed sequence tags (ESTs) that any omega-5 chain-terminating gliadins are expressed in Butte 86 [13]. Additionally, proteins with omega-5 gliadin-like sequences have not been detected in gluten polymer fractions prepared from flour of this cultivar [14]. In comparison, a number of omega-1,2 gliadins containing cysteine residues have been identified in Butte 86 by EST analysis [13] and the corresponding proteins are accumulated preferentially in soluble polymer fractions of Butte 86 flour [14].

Recently, RNA interference was used to silence the expression of genes encoding omega-5 gliadins in transgenic Butte 86 plants [15]. Two transgenic lines were selected in which the omega-5 gliadins either were significantly reduced or undetectable with minimal changes in the levels of other gluten proteins [16]. In the current work, these lines were grown in a greenhouse under a moderate temperature regimen with and without post-anthesis fertilizer and quantitative 2-DE was used to determine changes in the proteome that occur in response to the fertilizer treatment. Mixing and baking studies were performed on the same samples to determine the role of the omega-5 gliadins in flour quality. The main goals of this work were to determine whether it is possible to reduce or eliminate an important food allergen from wheat flour without having a negative impact on flour end-use quality (functionality) and to determine how the grain responds to the application of post-anthesis fertilizer in the absence of the omega-5 gliadins.

## Results

### Grain development in transgenic lines under different fertilizer regimens

Non-transgenic Butte 86 and transgenic lines SA-8-35b-5 and SA-8-45a-2, referred to here as 35b and 45a, were grown in controlled temperature greenhouses with and without post-anthesis fertilizer (20:20:20 NPK) and average grain weights were determined at 7, 14, 21, 28, 35 and 42 days post-anthesis (DPA). Maximum fresh weights for all samples were achieved at 35 DPA. For plants grown without post-anthesis fertilizer, the maximum fresh weights ranged from 78 to 81 mg/grain. When plants were supplied with post-anthesis fertilizer, the maximum fresh weights were slightly greater and ranged from 85 to 93 mg/grain. Bushel weights of mature grain averaged 64.8 +/− 0.3 lb/bu when plants were grown without post-anthesis fertilizer and 63.2 +/− 0.4 with fertilizer. There were no significant differences between the bushel weights of grain from the control and transgenic lines under either regimen. However, there were notable differences in the appearances of mature kernels produced under the two regimens. As shown in Additional file 1, samples from both the control and transgenic lines produced with post-anthesis fertilizer had uniform kernels with a vitreous appearance while those produced without post-anthesis fertilizer contained high percentages of yellow berries.

### Quantitative 2-DE analysis of flour protein composition in transgenic lines

**Figure 1:**
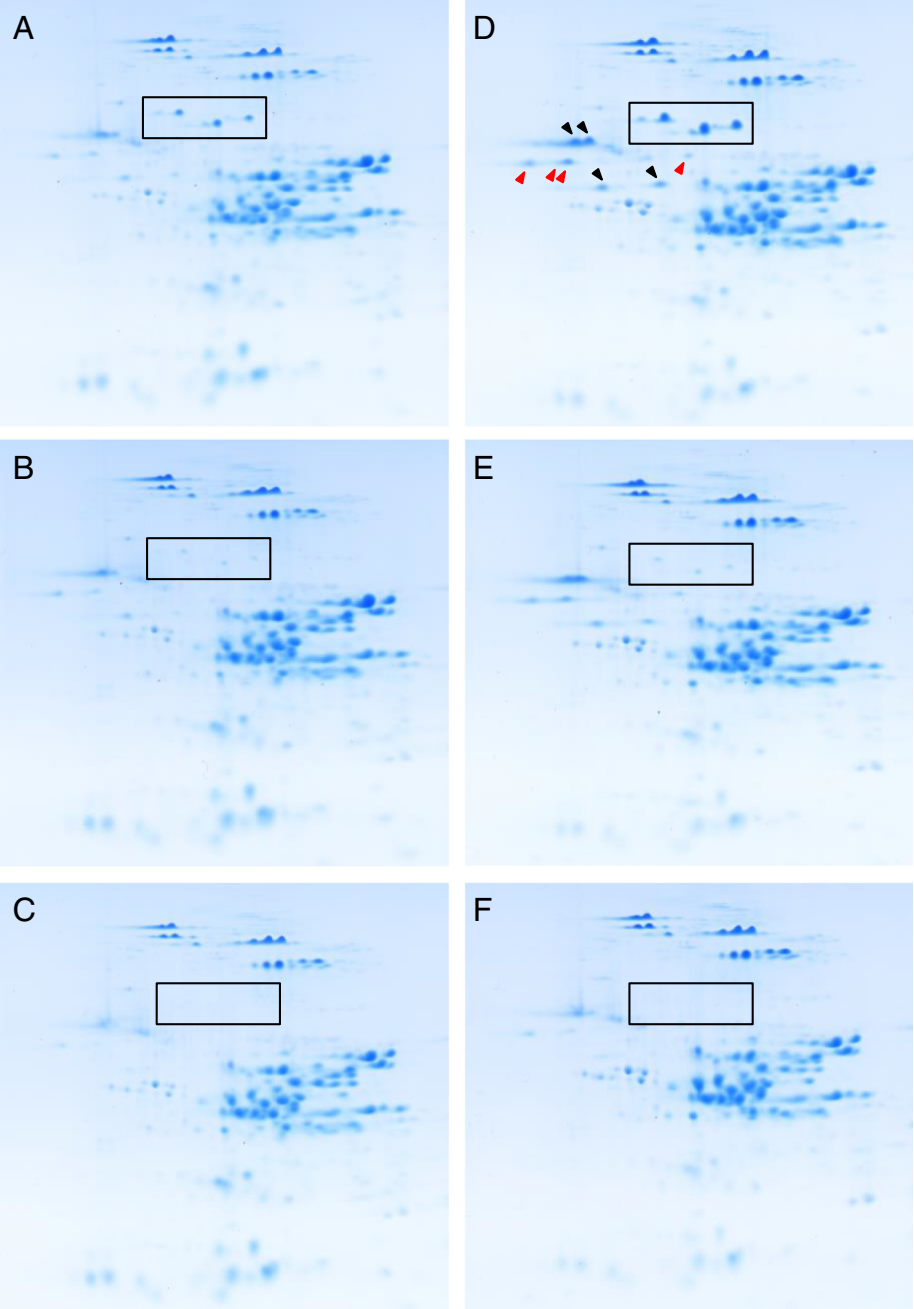
**Representative 2-D gels of flour proteins from non-transgenic and transgenic lines grown under different fertilizer regimens.** Flour proteins were from non-transgenic **(A, D)** and transgenic lines 35b **(B, E)** and 45a **(C, F)**. Flour in panels A-C was from plants grown without post-anthesis fertilizer while that in panels D-F was from plants supplied with post-anthesis fertilizer. Boxes enclose six protein spots identified previously as omega-5 gliadins. In panel D, spots identified as omega-1,2 gliadins and omega-1,2 chain-terminating gliadins are indicated with black and red arrowheads, respectively.

Total protein was extracted from white flour milled from triplicate samples of the three lines produced under the two fertilizer regimens and analyzed by 2-DE in triplicate. Representative gels are shown in Figure 1. The most striking differences between the control and the transgenic lines were evident in regions of the gels containing the omega gliadins. Six 2-DE spots previously identified as omega-5 gliadins in non-transgenic Butte 86 flour [6] (shown in boxes) were significantly less abundant in flour from transgenic line 35b (Figure 1B, E) than in the non-transgenic control (Figure 1A, D) and absent in flour from transgenic line 45a (Figure 1C, F), even with 3-D enhancement of gel images. As shown previously, greater amounts of omega-5 gliadins were accumulated in flour from non-transgenic Butte 86 plants supplied with post-anthesis fertilizer (Figure 1D) than in those that did not receive fertilizer (Figure 1A) [7,8]. Differences also were apparent in the relative amounts of four 2-DE spots identified as omega-1,2 gliadins (indicated with black arrowheads in Figure 1D) and four spots identified as omega-1,2 chain-terminating gliadins (indicated with red arrowheads in Figure 1D), especially between non-transgenic Butte 86 grown in the absence or presence of post-anthesis fertilizer (Figure 1A, D) as well as between the non-transgenic control and transgenic line 45a grown either without fertilizer (Figure 1A, C) or with fertilizer (Figure 1D, F).

**Figure 2:**
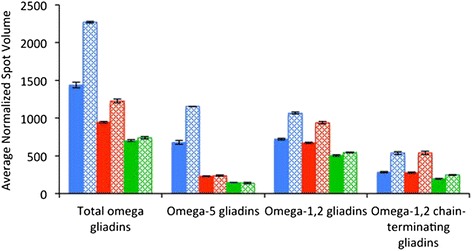
**Average normalized spot volumes of omega gliadins in flour from non-transgenic and transgenic lines grown under different fertilizer regimens.** For each class of proteins, the solid bars denote flour from plants grown without fertilizer while the stippled bars denote flour from plants supplied with post-anthesis fertilizer. Blue bars represent the non-transgenic control while red and green bars represent transgenic lines 35b and 45a, respectively. Total omega gliadins include the omega-5 gliadins, omega-1,2 gliadins and secalin-like omega gliadins, but not the omega-1,2 chain-terminating gliadins.

Volumes of individual 2-DE spots determined from each of the technical replicate gels were averaged and the average spot volumes were then determined for the biological replicates (Additional file 2). These values were summed for all spots previously identified as the same protein by tandem mass spectrometry [6] (Table 1). Accumulation of total omega gliadins, omega-5 gliadins, omega-1,2 gliadins, and omega-1,2 chain-terminating gliadins in flour from each of the lines produced under the two fertilizer regimens is summarized in Figure 2.

**Table 1:** **Changes in average normalized spot volumes of flour proteins in transgenic lines relative to the non-transgenic control grown with and without post-anthesis fertilizer**

		**# 2-DE spots that decrease** ^**1**^				**# 2-DE spots that increase** ^**1**^				**Average spot volume** ^**2**^						**% Change** ^**3**^			
		**Minus fertilizer**		**Plus fertilizer**		**Minus fertilizer**		**Plus fertilizer**		**Minus fertilizer**			**Plus fertilizer**			**Minus fertilizer**		**Plus fertilizer**	
**# Spots** ^4^	**Protein Identification** ^5^	**35b**	**45a**	**35b**	**45a**	**35b**	**45a**	**35b**	**45a**	**Control**	**35b**	**45a**	**Control**	**35b**	**45a**	**35b**	**45a**	**35b**	**45a**
8	HMW-GS Ax2^*^									478.37	478.88	501.45	561.18	557.40	570.27	0.1	4.8	−0.7	1.6
8	HMW-GS Bx7									824.00	850.20	871.81	968.99	987.02	938.06	3.2	5.8	1.9	−3.2
8	HMW-GS By9						3		1	417.26	431.28	505.14	550.88	583.14	635.55	3.4	**21.1**	5.9	15.4
8	HMW-GS Dx5		3	2	4					755.94	722.52	652.04	848.64	822.21	684.83	−4.4	−13.7	−3.1	−19.3
7	HMW-GS Dy10						1		1	594.53	597.64	650.34	702.68	707.96	751.93	0.5	9.4	0.8	7.0
39	**Total HMW-GS**									3070.10	3080.52	3180.79	3632.37	3657.73	3580.64	0.3	3.6	0.7	−1.4
2	LMW-GS Bu-1 (m-type)^6^									625.10	600.98	646.79	647.11	629.87	653.32	−3.9	3.5	−2.7	1.0
4	LMW-GS Bu-2/13 (s-type)^6^				2					551.57	552.98	493.66	638.08	640.46	532.08	0.3	−10.5	0.4	−16.6
8	LMW-GS Bu-3 (s-type)^6^	1	4	1	3					1266.56	1275.58	1061.02	1264.48	1249.93	1048.88	0.7	−16.2	−1.2	−17.1
3	LMW-GS Bu-4 (i-type)^6^									353.30	380.21	403.80	377.56	380.24	405.58	7.6	4.3	0.7	7.4
1	LMW-GS Bu-6 (m-type)^6^									254.17	253.36	249.13	226.14	214.33	208.26	−0.3	−2.0	−5.2	−7.9
3	LMW-GS Bu-7 (m-type)^6^			1						523.29	534.42	572.33	499.02	497.83	521.43	2.1	9.4	−0.2	4.5
1	LMW-GS Bu-8 (m-type)^6^									32.70	31.85	28.07	37.40	31.53	37.11	−2.6	−14.2	−15.7	−0.8
1	LMW-GS Bu-11 (m-type)^6^									124.72	132.19	140.70	107.97	108.53	110.90	6.0	2.8	0.5	2.7
2	LMW-GS Bu-18 (m-type)^6^									270.35	293.55	326.37	255.90	265.262	56.40	8.6	***20.7***	3.7	0.2
1	LMW-GS TC11-277270 (m-type)^6^		1		1					150.06	150.51	149.04	149.07	167.17	138.69	0.3	−0.7	12.1	−7.0
1	LMW-GS [GenBank: AAB48469] (i-type)^6^									60.73	54.40	34.03	68.93	67.85	40.05	−10.4	−**44.0**	−1.6	**−41.9**
27	**Total traditional LMW-GS**									4212.57	4260.01	4104.93	4271.68	4253.01	3952.71	1.1	−2.6	−0.4	−7.5
2	Alpha chain-terminating gliadin Bu-2^7^						1		1	402.61	410.38	451.61	451.36	455.88	485.50	1.9	12.2	1.0	7.6
3	Gamma chain-terminating gliadin Bu-4^8^		2	1	1					476.24	481.89	415.65	473.78	478.97	430.01	1.2	−12.7	1.1	−9.2
4	Omega-1,2 chain-terminating gliadin^9^		3		4					283.38	277.98	195.36	536.24	537.38	246.83	−1.9	**−31.1**	0.2	**−54.0**
9	**Total chain-terminating gliadins**									1162.23	1170.26	1062.62	1461.38	1472.22	1162.33	0.7	−8.6	0.7	**−20.5**
1	Alpha gliadin Bu-1									68.09	68.56	74.33	81.37	69.73	85.50	0.7	9.2	−14.3	5.1
1	Alpha gliadin Bu-3									180.74	182.36	204.19	210.70	221.22	232.06	0.9	13.0	5.0	10.1
1	Alpha gliadin Bu-4									306.35	313.67	349.35	353.82	354.76	367.54	2.4	4.0	0.3	3.9
2	Alpha gliadin Bu-5						1			190.87	209.23	239.66	285.97	296.50	312.78	9.6	**25.6**	3.7	9.4
1	Alpha gliadin Bu-10									196.26	206.54	197.39	208.34	214.01	201.55	5.2	0.6	2.7	−3.3
1	Alpha gliadin Bu-11									205.24	212.00	245.84	272.06	279.76	303.77	3.3	19.8	2.8	11.7
4	Alpha gliadin Bu-12						1			506.20	511.08	617.13	698.95	707.77	784.63	1.0	**21.9**	1.3	12.3
4	Alpha gliadin Bu-14						2	1	3	271.13	289.98	348.01	358.25	384.61	446.39	7.0	**28.4**	7.4	**24.6**
1	Alpha gliadin Bu-23									290.26	297.86	277.91	336.09	340.86	330.65	2.6	−4.3	1.4	−1.6
1	Alpha gliadin Bu-27	1		1			1			114.68	50.80	155.69	142.19	41.25	169.12	**−55.7**	**35.8**	**−71.0**	18.9
1	Alpha gliadin Bu-BQ806209									80.78	81.85	73.91	74.03	66.24	3.0	4.4	0.2	−10.4	80.78
1	Alpha gliadin Bu-BQ807130									96.61	107.05	114.95	125.15	119.48	135.56	10.8	19.0	−4.5	8.3
19	**Total alpha gliadin**									2504.83	2529.92	2906.30	3146.79	3103.98	3435.79	1.0	16.0	−1.4	9.2
2	Gamma gliadin Bu-1		1		2					205.88	217.38	179.18	223.09	212.75	176.47	5.6	−13.0	−4.6	**−20.9**
1	Gamma gliadin Bu-2									298.32	306.62	266.99	337.45	332.72	295.21	2.8	−10.5	−1.4	−12.5
4	Gamma gliadin Bu-5		1							795.04	816.60	738.76	798.55	802.41	719.11	2.7	−7.1	0.5	−9.9
3	Gamma gliadin Bu-6				2					294.02	308.72	291.19	341.59	321.44	282.37	5.0	−1.0	−5.9	−17.3
1	Gamma gliadin Bu-7									109.93	115.62	119.32	117.19	110.12	126.22	5.2	8.5	−6.0	7.7
2	Gamma gliadin Bu-11									281.31	285.23	300.08	303.39	296.76	315.99	1.4	6.7	−2.2	4.2
13	**Total gamma gliadin**									1984.50	2050.18	1895.51	2121.27	2076.21	1915.37	3.3	−4.5	−2.1	−9.7
6	Omega-5 gliadin	6	6	6	6					675.82	229.83	146.27	1153.93	237.46	140.56	**−66.0**	**−78.4**	**−79.4**	**−87.8**
2	Omega-1,2 gliadin D3^10^		1		2					479.80	458.24	393.76	631.49	581.38	410.86	−4.5	−17.9	−7.9	**−34.9**
2	Omega-1,2 gliadin Bu-D5^10^	1	2	1	2					242.54	214.06	111.87	435.25	355.39	133.28	−11.7	**−53.9**	−18.3	**−69.4**
1	Secalin-like omega gliadin									37.73	41.88	49.08	49.63	52.13	56.00	11.0	***30.1***	5.1	12.8
11	**Total omega gliadin**									1435.89	944.01	700.98	2270.30	1226.37	740.69	**−34.3**	**−51.2**	**−46.0**	**−67.4**
4	**Total mixed gliadin**									404.96	425.58	424.86	432.88	460.34	472.80	51	4.9	6.3	9.2
7	**Total triticin**						1		3	673.65	727.12	794.69	777.11	839.56	964.18	7.9	18.0	8.0	**24.1**
1	Farinin Bu-1									58.89	55.48	68.66	41.31	33.45	39.08	−5.8	16.6	−19.0	−5.4
3	Farinin Bu-2									334.76	320.74	307.56	231.53	221.26	221.18	−4.2	−8.1	−4.4	−4.5
2	Farinin Bu-3									143.03	154.74	146.48	125.87	124.27	128.67	8.2	2.4	−1.3	2.2
6	**Total farinin**									536.68	530.95	522.70	398.71	378.98	388.94	−1.1	2.6	−4.9	2.5
2	Purinin Bu-1						1			204.59	217.28	239.48	180.15	162.16	175.04	6.2	17.1	−10.0	−2.8
2	Purinin Bu-2				1					213.30	224.44	247.53	185.90	174.15	168.17	5.2	16.0	−6.3	−9.5
2	Purinin Bu-3									457.02	475.77	529.57	423.85	423.25	400.29	4.1	15.9	−0.1	−5.6
6	**Total purinin**									874.92	917.50	1016.58	789.89	759.56	743.49	4.9	16.2	−3.8	−5.9
1	Globulin-1									41.35	41.57	52.98	58.73	58.46	70.51	0.5	***28.1***	−0.5	***20.1***
5	Globulin-2	1	2	2	2					115.61	105.93	102.16	104.84	98.49	91.10	−8.4	−11.6	−6.1	−13.1
4	Globulin Glo-3		1		1					246.03	257.17	215.45	210.61	225.19	187.46	4.5	−12.4	6.9	−11.0
10	**Total globulin**									402.98	404.67	370.59	374.18	382.14	349.08	0.4	−8.0	2.1	−6.7
4	Serpin Bu-1						3			168.10	162.31	211.52	251.48	253.04	278.30	−3.4	**25.8**	0.6	10.7
2	Serpin Bu-2						2			53.48	52.28	70.47	108.94	114.09	119.34	−2.2	**31.8**	4.7	9.6
2	Serpin Bu-3								1	41.23	40.67	50.63	63.64	66.61	81.15	−1.4	**22.8**	4.7	**27.5**
1	Serpin Bu-4						1			67.65	69.35	83.86	105.73	111.12	128.48	2.5	***24.0***	5.1	***21.5***
2	Serpin Bu-5									83.63	83.32	103.75	126.05	125.89	152.76	−0.4	**24.1**	−0.1	**21.2**
2	Serpin Bu-7	1	1	1	1	1	1	1	1	59.27	62.64	63.17	74.88	70.10	73.42	5.7	6.6	−6.4	−2.0
13	**Total serpin**									473.36	470.56	583.41	730.71	740.86	833.45	−0.6	**23.2**	1.4	14.1
2	Beta-amylase Bu-2									102.36	93.67	101.60	99.71	98.17	99.37	−8.5	−0.7	−1.5	−0.3
3	Beta-amylase Bu-3	1	2	3	1					117.39	99.37	100.84	136.55	87.79	98.43	−15.4	−14.1	**−35.7**	**−27.9**
5	**Total beta-amylase**									219.75	193.03	202.43	236.27	185.97	197.80	−12.2	−7.9	−21.3	−16.3
2	WMAI Bu-1									624.43	660.27	661.82	469.28	488.91	456.32	5.7	6.0	4.2	−2.8
1	WDAI Bu-1									616.66	621.14	659.08	506.27	464.64	489.27	0.7	6.9	−8.2	−3.4
1	WDAI Bu-3									256.36	259.57	294.40	245.10	230.20	273.67	1.3	14.8	−6.1	11.7
1	WDAI Bu-4									179.79	194.48	206.86	130.61	113.52	126.49	8.2	15.1	−13.1	−3.1
1	WTAI CM1									223.52	233.25	247.31	183.44	172.34	170.34	4.3	10.6	−6.1	−7.1
2	WTAI CM2			1						1028.76	1046.46	1023.65	760.84	682.30	707.44	1.7	−0.5	−10.3	−7.0
2	WTAI CM3									653.13	660.93	658.76	483.08	436.22	402.44	1.2	0.9	−9.7	−16.7
2	WTAI CM16			1	1					549.43	517.83	535.74	397.30	332.88	337.40	−5.8	−2.5	−16.2	−15.1
2	WTAI CM17									442.72	437.00	443.39	307.31	263.48	266.70	−1.3	0.1	−14.3	−13.2
2	WCI									402.00	420.67	403.68	281.27	260.35	243.91	4.6	0.4	−7.4	−13.3
2	CMX1/CMX3									256.24	268.07	268.12	197.36	191.19	184.98	4.6	4.6	−3.1	−6.3
1	WASI									250.63	248.99	262.19	177.70	184.67	188.80	−0.7	4.6	3.9	6.2
19	**Total amylase/protease inhibitors**									5483.67	5568.66	5664.99	4139.56	3820.69	3847.76	1.5	3.3	−7.7	−7.0
32	**Other identified proteins**		1		1	1				2056.00	2108.86	2064.67	1888.87	1871.32	1966.62	2.6	0.4	−0.9	4.1

^1^Number of spots identified as each protein type that increased or decreased at least 20% in transgenic lines relative to non-transgenic control. Spot numbers and identities of all 2-DE spots that change can be found in Additional file 3.

^2^Sum of average volumes of all spots with the same MS/MS identification. MS/MS identifications are from [6].

^3^Protein types that that increased or decreased at least 20% in transgenic lines relative to non-transgenic control are indicated in bold. Changes that were greater than 20% but were not deemed to be significant by ANOVA are shown in bold italics.

^4^Total number of spots in 2-D gels that were identified as the same protein type in [6].

^5^Predominant protein in spots identified by MS/MS in [6]. Protein classes are indicated in bold type.

^6^Classification of traditional LMW-GS as m-type, s-type and i-type is based on the first amino acid of the mature proteins; methionine, serine or isoleucine.

^7^Protein has amino acid sequence similar to an alpha gliadin but contains seven cysteines that enables it to be linked into the glutenin polymer.

^8^Protein has amino acid sequence similar to a gamma gliadin but contains nine cysteines that enables it to be linked into the glutenin polymer.

^9^Protein has amino acid sequence similar to an omega-1,2 gliadin but contains one cysteine that enables it to be linked into the glutenin polymer.

^10^Two distinct protein types were distinguished for omega-1,2 gliadins. Percent changes of total omega-1,2 gliadins are −6.9 and −30.0 for 35b and 45a grown without fertilizer, respectively, and −12.2 and −49.0 for 35b and 45a grown with fertilizer, respectively.

In the absence of post-anthesis fertilizer, total omega gliadins were reduced 34% in transgenic line 35b and 51% in transgenic line 45a relative to the non-transgenic control (Table 1, Figure 2). When plants received fertilizer, the reductions in total omega gliadins were even greater, 46% for 35b and 67% for 45a. In line 35b, reductions were due almost entirely to suppression of the omega-5 gliadins. In line 45a, the reductions were due to suppression of the omega-5 gliadins as well as partial suppression of the omega-1,2 gliadins and the omega-1,2 chain-terminating gliadins (Figure 2).

The omega-5, omega-1,2 and omega-1,2 chain-terminating gliadins increased 71%, 48% and 89% when the non-transgenic plants were supplied with post-anthesis fertilizer (Table 2, Figure 2). There was little change in the levels of omega-5 gliadins in transgenic line 35b in response to fertilizer. However, both the omega-1,2 and the omega-1,2 chain-terminating gliadins showed increases in this line of 39% and 93%, respectively. Since the omega-5 gliadins were not detectable in transgenic line 45a, the normalized volumes in Figure 2 represent the sums of background values for the six omega-5 gliadin spots. There was little change in the levels of omega-1,2 gliadins and only a small increase in the levels of the omega-1,2 chain-terminating gliadins in this line.

**Table 2:** **Specific flour protein types that increased or decreased in relative amounts when non-transgenic and transgenic plants were grown with post-anthesis fertilizer**

	**%Change with Fertilizer** ^**1**^		
**Protein Type** ^**2**^	**Control**	**35b**	**45a**
HMW-GS By9	**32.0**	**35.2**	**25.8**
LMW-GS Bu-11 (m-type)	−13.4	−17.9	**−21.2**
LMW-GS Bu-18 (m-type)	−5.3	−9.6	−**21.4**
LMW-GS [GenBank: AAB48469] (i-type)	13.5	**24.7**	17.7
Omega-1,2 chain-terminating gliadin^3^	**89.2**	**93.3**	**26.3**
Alpha gliadin Bu-5	**49.8**	**41.7**	**30.5**
Alpha gliadin Bu-11	**32.6**	**32.0**	**23.6**
Alpha gliadin Bu-12	**38.1**	**38.5**	**27.1**
Alpha gliadin Bu-14	**32.1**	**32.6**	**28.3**
Alpha gliadin Bu-27	**24.0**	−18.8	8.6
Alpha gliadin Bu-BQ807130	**29.5**	11.6	17.9
Omega-5 gliadin	**70.7**	3.3	−3.9
Omega-1,2 gliadin D3^4^	**31.6**	**26.9**	4.3
Omega-1,2 gliadin Bu-D5^4^	**79.5**	**66.0**	19.1
Secalin-like omega gliadin	**31.5**	***24.5***	14.1
Triticin	15.4	15.5	**21.3**
Farinin Bu-1	**−29.9**	***−39.7***	**−43.1**
Farinin Bu-2	**−30.8**	**−31.0**	**−28.1**
Purinin Bu-1	−11.9	**−25.4**	**−26.9**
Purinin Bu-2	−12.8	**−22.4**	**−32.1**
Purinin Bu-3	−7.3	−11.0	**−24.4**
Serpin Bu-1	**49.6**	**55.9**	**31.6**
Serpin Bu-2	**103.7**	**118.2**	**69.3**
Serpin Bu-3	**54.3**	**63.8**	**60.3**
Serpin Bu-4	**56.3**	**60.2**	**53.2**
Serpin Bu-5	**50.7**	**51.1**	**47.2**
Serpin Bu-7	**26.3**	11.9	16.2
WMAI Bu-1	**−24.8**	**−26.0**	**−31.1**
WDAI Bu-1	−17.9	**−25.2**	**−25.8**
WDAI Bu-4	**−27.4**	**−41.6**	**−38.8**
WTAI CM1	−17.9	**−26.1**	**−31.1**
WTAI CM2	**−26.0**	**−34.8**	**−30.9**
WTAI CM3	**−26.0**	**−34.0**	**−38.9**
WTAI CM16	**−27.7**	**−35.7**	**−37.0**
WTAI CM17	**−30.6**	**−39.7**	**−39.8**
WCI	**−30.0**	**−38.1**	**−39.6**
CMX1/CMX3	**−23.0**	**−28.7**	**−31.0**
WASI	**−29.1**	**−25.8**	**−28.0**

^1^Protein types that either increased or decreased at least 20% in response to post-anthesis fertilizer are indicated in bold. Changes greater than 20% that were not deemed to be significant by ANOVA are shown in bold italics.

^2^Only protein types that changed in response to fertilizer in at least one line are shown.

^3^Protein has an amino acid sequence similar to an omega-1,2 gliadin but contains one cysteine that enables it to be linked into the glutenin polymer.

^4^Two distinct protein types were distinguished for omega-1,2 gliadins. Percent changes of total omega-1,2 gliadins with fertilizer were 47.7, 39.3 and 7.6 for the non-transgenic, 35b and 45a, respectively.

In addition to the changes in the accumulation of omega gliadins in transgenic line 35b, five individual 2-DE spots showed small, but significant decreases in the absence of fertilizer relative to the control (Table 1; Additional file 3) while two spots showed small, but significant increases (Additional file 3). When the same transgenic line was supplied with post-anthesis fertilizer, 14 spots showed decreases and two spots showed increases relative to the non-transgenic control. Transgenic line 45a exhibited a greater number of changes in individual spots. In addition to 12 omega gliadin spots that decreased when plants were grown without fertilizer, 19 spots showed significant decreases (Table 1; Additional file 3) and 19 other spots showed significant increases (Table 1; Additional file 3). When this transgenic line was supplied with fertilizer, 23 individual spots in addition to the 14 omega gliadin spots showed small, but significant decreases (Table 1; Additional file 3) and 11 spots showed increases relative to the control (Table 1; Additional file 3).

When the normalized volumes of all spots with the same MS/MS identification were summed (Table 1), the analysis revealed that very few proteins changed in abundance in line 35b other than the omega-5 gliadins. A minor alpha gliadin Bu-27 decreased 56% and 71% in plants grown in the absence and presence of post-anthesis fertilizer, respectively, while beta-amylase Bu-3 decreased 36% in flour from plants grown with fertilizer. In transgenic line 45a excepting the omega gliadins and the omega chain-terminating gliadins, there were decreases in a minor i-type LMW-GS in flour from plants grown under both fertilizer regimens as well as small decreases in gamma gliadin Bu-1 and beta-amylase Bu-3 in plants supplied with fertilizer. However, there were also increases in a number of different proteins in 45a. In the absence of post-anthesis fertilizer, HMW-GS By9, alpha gliadins Bu-5, Bu-12, Bu-14 and Bu-27, and serpins Bu-1, Bu-2, Bu-3, and Bu-5 increased from 21 to 36%. When plants were supplied with post-anthesis fertilizer, there were increases in only alpha gliadin Bu-14, serpins Bu-3 and Bu-5, and triticin.

Overall, the only protein class that changed in transgenic line 35b in the absence of fertilizer was the omega gliadin class, while decreases in the omega gliadin class were accompanied by a small increase in the serpins in line 45a. When plants were supplied with fertilizer, there were decreases in both the omega gliadins and the beta-amylases in 35b. In line 45a supplied with fertilizer, there were decreases in the omega gliadins and the chain-terminators, largely due to decreases in the omega-1,2 chain-terminating gliadins, as well as increases in the triticins.

### Effect of post-anthesis fertilizer on flour protein composition

In the non-transgenic plants, the omega gliadins and the chain-terminating omega gliadins exhibited some of the largest responses to post-anthesis fertilizer (Table 2). There were also notable increases in a number of serpins, a subset of alpha gliadins and HMW-GS By9. There were decreases in several farinins and most alpha-amylase/protease inhibitors. With the exception of the omega gliadins, most of the same proteins that responded to fertilizer in the non-transgenic control exhibited a similar response to fertilizer in the two transgenic lines. Among the gluten proteins, there were increases in HMW-GS By9 and alpha gliadins Bu-5, Bu-11, Bu-12 and Bu-14. Most serpins also increased with post-anthesis fertilizer and there was a small increase in a minor LMW-GS in 35b and triticin in 45a while most alpha amylase/protease inhibitors, and some farinins and purinins decreased in both transgenic lines. Small decreases in two m-type LMW-GS also were noted in 45a.

Ratios of HMW-GS to LMW-GS determined from the spot volume data were 0.57, 0.57 and 0.62 when non-transgenic, 35b and 45a plants were grown without post-anthesis fertilizer, respectively, and 0.63, 0.64 and 0.70 when non-transgenic, 35b and 45a plants received fertilizer, respectively (Additional file 4). The ratios of gliadins to glutenins were 0.75, 0.70 and 0.71 when non-transgenic, 35b and 45a plants were grown without post-anthesis fertilizer, respectively, and 0.85, 0.73 and 0.75 when non-transgenic, 35b and 45a plants received fertilizer, respectively (Additional file 4).

### End-use quality of transgenic lines

**Figure 3:**
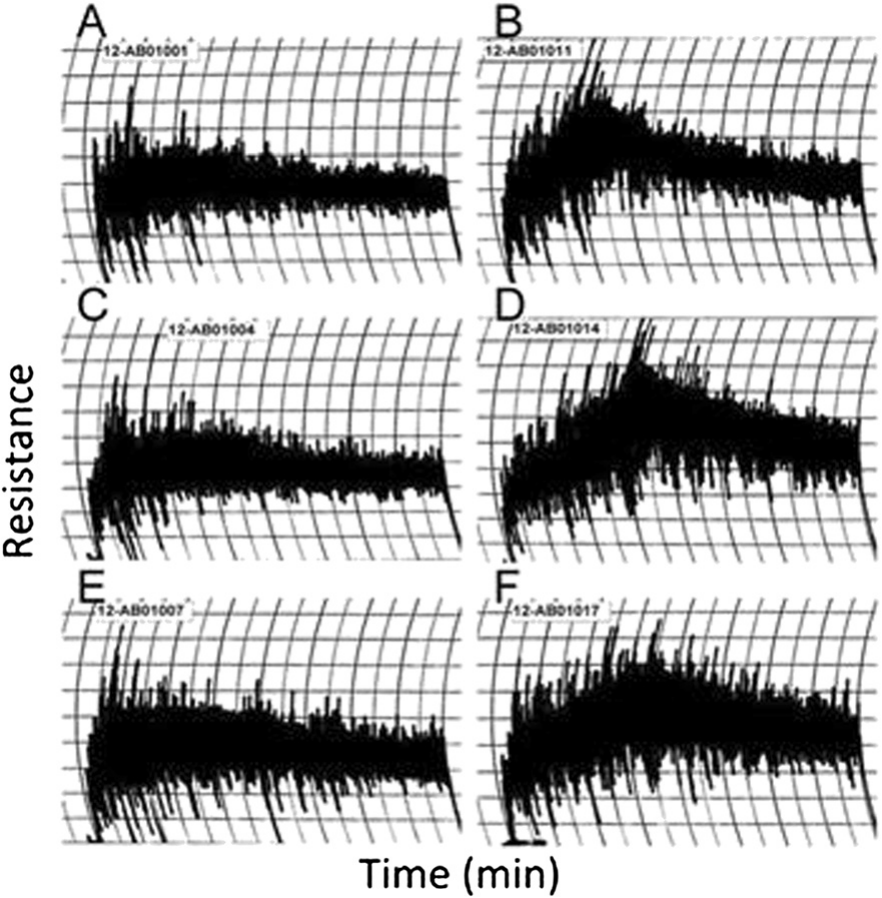
**End-use quality of flour from non-transgenic and transgenic lines grown under different fertilizer regimens.** Mixograms produced with flour from non-transgenic **(A, B)** and transgenic lines 35b **(C, D)** and 45a **(E, F)** grown without **(A, C, E)** and with **(B, D, F)** post-anthesis fertilizer. For each trace, time (min) is shown on the x-axis and resistance is shown on the y-axis.

There was little difference between the protein content of flour produced from the non-transgenic and transgenic plants (Table 3). Flour from plants grown without post-anthesis fertilizer had average protein contents of 9.0% (STD = 0.3) while those from plants grown with post-anthesis fertilizer averaged 16.0% (STD = 0.6). Representative mixograms (mixograph curves) are shown in Figure 3. The corrected mix times for flour from non-transgenic Butte 86 were 1.6 and 2.4 min for plants grown without and with post-anthesis fertilizer, respectively. Mix time was little changed for flour from transgenic plants grown without fertilizer, but increased to 3.5 and 3.6 min for 35b and 45a, respectively, when plants were grown with post-anthesis fertilizer. Mixing tolerance (resistance to over-mixing) also increased in flour from transgenic plants, particularly when grown with post-anthesis fertilizer. Mixing tolerance (0–6 scale) was 2.3 for the non-transgenic plant grown with fertilizer and 4.0 for both of the transgenic lines. As shown previously for Butte 86, loaf volumes correlated with protein contents of the flour [17,18] and averaged 683.3 cc for the non-transgenic control when plants were grown without fertilizer and 928.3 cc when plants were grown with fertilizer. A 5% increase in loaf volume was observed in 35b relative to the control when plants were grown with fertilizer. For the non-transgenic flours, SDS sedimentation volumes also were related to protein contents and averaged 29.3 ml without fertilizer and 63.1 ml with fertilizer. SDS sedimentation volumes were about 5% higher for 35b and 10% higher for 45a.

**Table 3:** **End-use quality data for flour from non-transgenic and transgenic lines**

**Plant**	**Treatment**	**Flour protein (%)** ^**1,2**^	**Corrected mix time (min)** ^**1**^	**Mix tolerance** ^**1,3**^	**Loaf volume (cc)** ^**1**^	**SDS sedimentation (ml)** ^**1**^
Non-transgenic	Minus Fertilizer	9.1 (0.3)	1.6 (0.2)	2.0 (0.0)	683.3 (25.2)	29.3 (0.7)
Transgenic 35b	Minus Fertilizer	8.7 ( 0.1)	1.5 (0.4)	2.3 (0.6)	678.3 (5.8)	30.7 (0.3)
Transgenic 45a	Minus Fertilizer	9.2 (0.2)	1.9 (0.1)	3.0 (0.0)	715.0 (17.3)	32.4 (0.5)
Non-transgenic	Plus Fertilizer	16.0 (0.2)	2.4 (0.2)	2.3 (0.6)	928.3 (20.2)	63.1 (0.3)
Transgenic 35b	Plus Fertilizer	16.5 (0.2)	3.5 (0.3)	4.0 (0.0)	976.7 (2.9)	66.4 (0.1)
Transgenic 45a	Plus Fertilizer	15.4 (0.4)	3.6 (0.2)	4.0 (0.0)	923.3 (2.9)	69.4 (0.1)

^1^Averages and (standard deviations) from flour samples from three biological replicates are reported. ^2^Based on 14% moisture. ^3^Recorded on a 0-6 scale with 6 having the greatest tolerance.

## Discussion

Analysis of transgenic lines in which omega-5 gliadin genes were suppressed by RNA interference provides new insight into the roles of these proteins in flour end-use quality. Key to this work is the creation of wheat lines in which only a specific set of proteins has been altered. In the current study, a detailed proteomic analysis of flour from transgenic line 35b revealed that there were very few changes in the levels of proteins other than the omega-5 gliadins that were targeted by the RNAi construct. Flour protein content was determined by the fertilizer regimen and was similar in both the transgenic line and the control. However, flour functionality was improved in the transgenic line when plants were supplied with post-anthesis fertilizer as evidenced by increases in mixing time, mixing tolerance and loaf volume. Thus, the data suggest that the omega-5 gliadins have a negative effect on the functionality of the flour. A second transgenic line, 45a, exhibited complete suppression of the omega-5 gliadins as well as partial suppression of the omega-1,2 gliadins. Because the omega 1,2 chain-terminating gliadins have very similar primary sequences to the omega-1,2 gliadins, there was also partial suppression of these proteins. A decrease in the chain-terminating gliadins might be expected to increase polymer size and improve flour end-use quality. Flour from line 45a also showed increased mixing time and tolerance relative to the control when plants were grown with post-anthesis fertilizer, although magnitudes of the changes were not much different than that observed in 35b.

The overall findings are in agreement with Waga and Skoczowski [19] who also suggested that elimination of omega gliadins improved flour end-use quality. However, they evaluated lines obtained using traditional plant breeding methods that exhibited numerous other differences in flour protein composition when analyzed by RP-HPLC and their conclusions were based solely on the results of SDS sedimentation tests. The fact that genes for omega gliadins, gamma gliadins and LMW-GS are closely linked and inherited as blocks makes it difficult to use genetic approaches to discern the effects of specific protein types on flour functionality. Transgenic approaches such as gene silencing by RNAi offer a way to target individual genes within these blocks. However, RNAi also can have off-target or compensatory effects on the proteome that confound the interpretation of results. Indeed there are numerous cases in the literature where suppression of a class of gluten proteins by RNAi results in considerable changes in the flour protein profile [20-25]. In one series of studies, the silencing of gamma gliadins in transgenic wheat was accompanied by increases in omega and alpha gliadins and some glutenins [21-23,25]. In another study, the silencing of alpha gliadins resulted in compensatory increases in omega and gamma gliadins, HMW-GS, and albumins/globulins [20]. Although some Butte 86 transgenic lines exhibited unintended effects of gene silencing [16], there were also lines in which the omega-5 gliadins were reduced or absent with minimal off-target or compensatory effects. By selecting these lines for detailed proteomic analyses and quality assessments it was possible to obtain convincing evidence that the omega-5 gliadins have a negative effect on end-use quality.

In transgenic line 35b, levels of omega-5 gliadins were modulated by RNAi under both fertilizer regimens. Although small amounts of omega-5 gliadins were detected in the flour when plants were grown in the absence of post-anthesis fertilizer, the levels did not increase when plants were supplied with fertilizer as was observed in flour from the non-transgenic plants. In comparison, the levels of omega-1,2 gliadins and the omega-1,2 chain-terminating gliadins, which were not suppressed in line 35b, increased in response to fertilizer, similar to the non-transgenic control. Previous studies using quantitative RT-PCR showed that transcript profiles for the omega-5 and omega-1,2 gliadin genes were very similar in Butte 86 grain and that both responded to post-anthesis fertilizer in a like manner [13]. In the absence of fertilizer under a 24/17°C temperature regimen, transcripts began to accumulate by 8 DPA, reached maximum levels by 22 DPA, and then declined at 26 and 32 DPA. With post-anthesis fertilizer, transcripts were first detected at 8 DPA and increased throughout grain development, reaching maximum levels at 32 DPA. Additionally, maximum levels of transcripts for both omega-5 gliadins and omega-1,2 gliadins were considerably higher in plants grown with fertilizer than without [13]. In the current study, the RNAi construct introduced into the transgenic plants to silence the omega-5 gliadin genes utilized the Dy10 HMW-GS promoter. Since HMW-GS transcripts also are first detectable by 8 DPA in grain from Butte 86 plants grown under a 24/17°C temperature regimen [26], the hairpin RNA produced by the RNAi construct in this study could initiate the silencing mechanism early in grain development and might be expected to interfere with the accumulation of omega-5 gliadin transcripts to a similar degree in grain produced under either fertilizer regimen. In transgenic line 45a, complete silencing of the omega-5 gliadins was accompanied by a partial reduction in the levels of the omega-1,2 gliadins and the omega-1,2 chain-terminating gliadins, probably an effect of off-target silencing [16]. Any off-target silencing also would be initiated early in grain development and would be expected to be similar in grain produced under either fertilizer regimen. It is likely that the levels of omega-5 gliadins accumulated in the grain were set by the amount of hairpin RNA expressed in the line. This opens up the possibility that RNA interference may be used to fix the levels of specific proteins in the flour so that they do not vary with environmental conditions, thereby making the overall functionality of the flour more stable and predictable.

The current work also provides insight into how protein composition of the grain is controlled. Flour protein contents did not change when the omega-5 gliadins were suppressed in the transgenic plants. It is noteworthy that the down-regulation of the sulfur-poor omega-5 gliadins did not result in a compensatory increase in other sulfur-poor proteins in the grain, even when plants were supplemented with post-anthesis fertilizer. To maintain the same level of protein in 35b, it is likely that small increases were distributed among most of the other flour proteins. The same is probably true in 45a. Although there were moderate increases in several alpha gliadins and serpins in this line, it is unlikely that these increases were sufficient to offset the suppression of the omega-5 gliadins and the partial suppression of the omega-1,2 gliadins and the omega-1,2 chain terminating gliadins. It is also interesting that, with the exception of the omega gliadins, most of the same proteins that showed changes in response to post-anthesis fertilizer in the control also showed changes in the transgenic lines.

Among the gluten proteins, the omega gliadins show some of the largest responses to fertilizer and high temperature. By suppressing the expression of the omega-5 gliadins in transgenic lines, one of the major sources of variation in flour protein composition has been eliminated. Since these proteins also appear to influence mixing properties, it is possible that the transgenic lines may yield flour that has more consistent functionality when grown under different environmental conditions. Field tests of the transgenic lines at a variety of different locations combined with analyses of end-use quality would determine whether the transgenic lines offer this additional advantage. In addition, similar studies specifically targeting the omega-1,2 gliadins are warranted since these proteins also show large changes in response to both fertilizer and high temperature.

## Conclusions

This study demonstrates that it is possible to eliminate a group of proteins that are a major source of environmental variability as well as an important food allergen from wheat flour without compromising flour functionality. Rather, flour from transgenic lines in which the omega-5 gliadins were significantly reduced or eliminated showed improved mixing properties, suggesting that the omega-5 gliadins have a negative effect on the functional properties of the flour. Other than the omega gliadins, changes in the flour proteome in response to the application of post-anthesis fertilizer were similar in non-transgenic and transgenic lines. It is thus likely that the RNAi approach to silence omega gliadin genes may result in plants with more consistent flour end-use quality under changing environmental conditions.

## Methods

### Growth of plants

Transgenic wheat plants produced by genetically transforming *T. aestivum* cv. Butte 86 with an RNAi construct targeting the omega-5 gliadins were reported previously [15]. Following a detailed proteomic analysis of transgenic grain, two homozygous lines were selected in which omega-5 gliadins were either significantly reduced or eliminated with minimal effects on the accumulation of other proteins [16]. These lines, SA-8-35b-5 and SA-8-45a-2, are referred to as 35b and 45a. The non-transgenic control and transgenic lines 35b and 45a were grown in 3 gal pots containing Sunshine Mix Number 1 (SunGro Horticulture, Inc. Bellevue, WA, USA) in a controlled temperature greenhouse under a 24/17°C day/night regimen as described previously [27]. Thirty-six pots, each containing seven plants, were grown for each line to ensure that enough grain was obtained for end-use quality analyses. Prior to anthesis, plants were supplied with Peter’s Professional 20-20-20 water-soluble fertilizer (Scotts-Sierra Horticultural Products Company, Marysville, OH) through a drip irrigation system. At anthesis, pots from each line were divided into six groups containing six pots each. Three sets of pots for each line were flushed with water to remove remaining fertilizer and subsequently hand-watered without fertilizer. The remaining three sets of pots for each line received 500 mls of 0.6 g/liter 20-20-20 fertilizer per day through a drip irrigation system. Fertilizer regimens were the same as in previous studies [7,8,17,18] and were selected to highlight differences in grain protein contents and composition rather than to mimic field conditions. All pots were weighed weekly and adjusted to 80% water capacity as necessary.

Heads were tagged at anthesis. Developing grain was harvested from three separate heads collected at 7, 14, 21, 28, 35 and 42 DPA from each line and treatment. Ten kernels were collected from the center of each head and average kernel weight was determined. At maturity, grain was harvested from the three biological replicates of each line grown under each fertilizer regimen. Grain samples (300 g) were sent to the Hard Winter Wheat Quality Laboratory (HWWQL) and milled to straight-grade flour using methods standardized by the American Association of Cereal Chemists (AACC Approved Methods 26–10.02 and 26–22.01) [28].

### Quantitative 2-DE analysis of flour protein composition

Total protein was extracted from 50 mg samples of flour from each of the biological replicates with SDS under reducing conditions as described previously [6]. Protein amount was determined using the method of Lowry et al. [29]. Proteins were analyzed by 2-DE in triplicate as described in detail previously [14]. The experimental design included three biological replicates, each with three technical replicates.

Gels were digitized using a calibrated scanner at 310 dpi (Epson Perfection V750 PRO, Long Beach, CA). 2-DE spots were matched between gels, quantified and normalized using SameSpots v. 4.5 (TotalLab, Ltd., Newcastle upon Tyne, UK). Five hundred ten spots were detected and quantified by the SameSpots software.

Comparisons were made between triplicate gels from each of the three biological replicates for non-transgenic and transgenic lines 35b and 45a under each growth regimen using the SameSpots software. Comparisons also were made between the two growth regimens for each line. Differentially expressed spots were identified using analysis of variance (ANOVA) performed by the SameSpots software. All differentially expressed spots had ANOVA values <0.01 and showed at least 1.2-fold changes.

Of the 510 spots analyzed, 220 of the most abundant protein spots had been identified previously from Butte 86 flour by MS/MS [6]. These spots accounted for between 77.2 and 79.0% of the total spot volume detected by the software. Upon manual inspection, each of the 220 spots was correlated with a protein spot from Dupont et al. [6]. The corresponding spot number from Dupont et al. [6] is provided as a reference spot number in Additional file 2 and Additional file 3 along with the identification of the predominant protein determined by MS/MS in the same study. Identifications of spots for alpha-amylase/protease inhibitors are from Altenbach et al. [30]. The average spot volumes of identified spots ranged from 112 to 121 for the six samples. The 290 other spots detected by the SameSpots software generally were very minor spots. Because their average volumes ranged from 23–25, these spots were excluded from further analysis. Spot volume data and statistical analyses are shown in Additional file 2. Individual spots that showed significant changes are presented in Additional file 3.

Average volumes of all spots identified as either HMW-GS or LMW-GS (traditional LMW-GS plus chain-terminating gliadins) were summed for each flour sample and used to determine the ratio of HMW-GS to LMW-GS in that sample. Likewise, average volumes of all spots identified as gliadin (alpha, gamma and omega) or glutenin (HMW-GS and LMW-GS) were summed and used to determine the ratio of gliadin to glutenin in that sample. Spots grouped in each gluten protein family are shown in Additional file 4 along with the relevant statistics.

### Flour end-use quality

End-use functionality tests (quality) were conducted on each of the biological replicates at the HWWQL (US Department of Agriculture, Agricultural Research Service, Manhattan, KS) using American Association of Cereal Chemists (AACC) approved methods that are routinely used for end-use quality assessment of breeding lines [28]. Protein content of flour was determined by NIR using AACC Method 39–11.01. Mixing properties (dough rheology) were determined on 10 g of flour from each sample using a Mixograph (National Mfg., Lincoln, NE) and AACC Method 54–40.02. Optimized straight dough bake tests were performed with 100 g flour using AACC Method 10–10.03. SDS sedimentation tests were conducted according to AACC Method 56–60.01. Averages and standard deviations were calculated for the biological replicates from each wheat line under each fertilizer regimen.

### Availability of supporting data

All supporting data for this manuscript are included in Additional files.

## Additional files

Additional file 1:
**Appearances of mature kernels from control and transgenic plants grown with and without post-anthesis fertilizer.**
Additional file 2:
**Statistical analyses of normalized spot volumes of flour proteins from non-transgenic control and transgenic lines SA-8-35b-5 and SA-8-45a-2 grown with and without post-anthesis fertilizer.**
Additional file 3:
**Individual 2-DE spots that show significant changes.**
Additional file 4:
**2-DE spots containing gluten proteins grouped by class.**

## Abbreviations

2-DE: Two-dimensional gel electrophoresis
DPA: Days post-anthesis
EST: Expressed sequence tags
MS/MS: Tandem mass spectrometry
HMW-GS: High molecular weight glutenin subunit
LMW-GS: Low molecular weight glutenin subunit
RNAi: RNA interference
RP-HPLC: Reverse-phase high pressure liquid chromatography
RT-PCR: Reverse-transcriptase polymerase chain reaction
WDEIA: Wheat-dependent, exercise-induced anaphylaxis
