# Supplementary material for: Silencing of omega-5 gliadins in transgenic wheat eliminates a major source of environmental variability and improves dough mixing properties of flour
Source: BMC Plant Biol. 2014 Dec 24;14:1. doi: 10.1186/s12870-014-0393-1 (PMC4307166; doi:10.1186/s12870-014-0393-1)
Supplement: Additional file 1: — Appearances of mature kernels from control and transgenic plants grown with and without post-anthesis fertilizer. [file 12870_2014_393_MOESM1_ESM.pptx]

## Slide 1
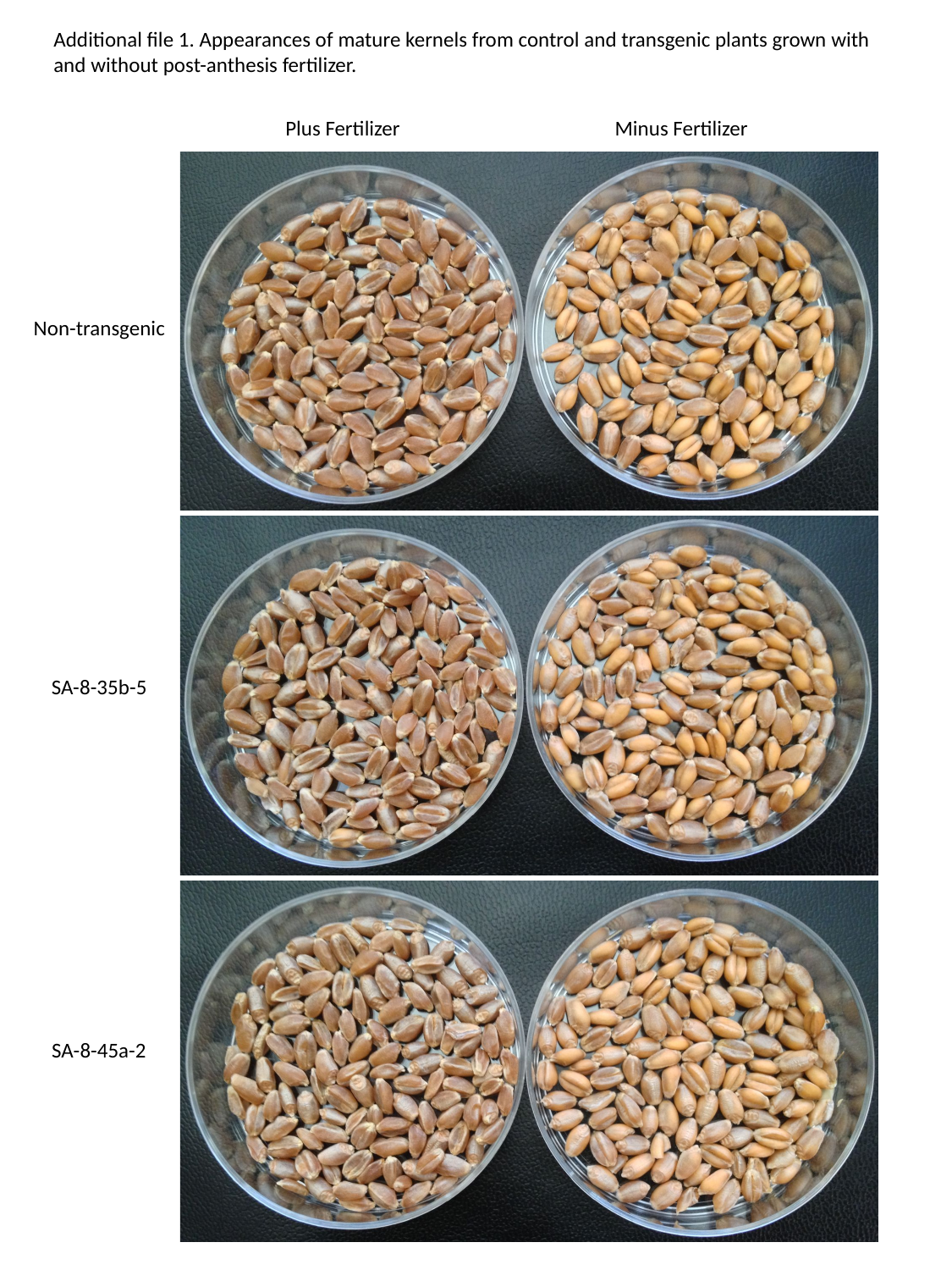

Additional file 1. Appearances of mature kernels from control and transgenic plants grown with and without post-anthesis fertilizer.
Plus Fertilizer
Minus Fertilizer
Non-transgenic
SA-8-35b-5
SA-8-45a-2
